# Pleiotropy of autism-associated chromatin regulators

**DOI:** 10.1242/dev.201515

**Published:** 2023-07-18

**Authors:** Micaela Lasser, Nawei Sun, Yuxiao Xu, Sheng Wang, Sam Drake, Karen Law, Silvano Gonzalez, Belinda Wang, Vanessa Drury, Octavio Castillo, Yefim Zaltsman, Jeanselle Dea, Ethel Bader, Kate E. McCluskey, Matthew W. State, A. Jeremy Willsey, Helen Rankin Willsey

**Affiliations:** ^1^ Department of Psychiatry and Behavioral Sciences, University of California, San Francisco, San Francisco, CA 94143, USA; ^2^ Weill Institute for Neurosciences, University of California, San Francisco, San Francisco, CA 94143, USA; ^3^ Langley Porter Psychiatric Institute, University of California, San Francisco, San Francisco, CA 94143, USA; ^4^ Quantitative Biosciences Institute, University of California, San Francisco, San Francisco, CA 94143, USA; ^5^ Chan Zuckerberg Biohub - San Francisco, San Francisco, CA 94158, USA

**Keywords:** Autism spectrum disorder, Microtubules, Tubulin, Spindles, Cilia, Chromatin, Cell cycle, *Xenopus*, Human

## Abstract

Gene ontology analyses of high-confidence autism spectrum disorder (ASD) risk genes highlight chromatin regulation and synaptic function as major contributors to pathobiology. Our recent functional work *in vivo* has additionally implicated tubulin biology and cellular proliferation. As many chromatin regulators, including the ASD risk genes *ADNP* and *CHD3*, are known to directly regulate both tubulins and histones, we studied the five chromatin regulators most strongly associated with ASD (*ADNP*, *CHD8*, *CHD2*, *POGZ* and *KMT5B*) specifically with respect to tubulin biology. We observe that all five localize to microtubules of the mitotic spindle *in vitro* in human cells and *in vivo* in *Xenopus*. Investigation of *CHD2* provides evidence that mutations present in individuals with ASD cause a range of microtubule-related phenotypes, including disrupted localization of the protein at mitotic spindles, cell cycle stalling, DNA damage and cell death. Lastly, we observe that ASD genetic risk is significantly enriched among tubulin-associated proteins, suggesting broader relevance. Together, these results provide additional evidence that the role of tubulin biology and cellular proliferation in ASD warrants further investigation and highlight the pitfalls of relying solely on annotated gene functions in the search for pathological mechanisms.

## Teaser

**Highlighted Article:** This work implicates five autism-associated chromatin regulators in mitotic spindle regulation and provides evidence for broader convergence of tubulin-related biology in autism risk.

## INTRODUCTION

The past two decades have yielded striking progress identifying high-confidence (hc), large-effect risk genes for autism spectrum disorders (ASD), with robust statistical support for over 200 genes (Fu et al., 2022; Willsey et al., 2022). Gene ontology analyses have repeatedly emphasized enrichment of terms related to synaptic biology and gene expression regulation by transcription factors and chromatin regulators (Satterstrom et al., 2020; De Rubeis et al., 2014; Neale et al., 2012; Iossifov et al., 2012; Sanders et al., 2012). At the same time, our hypothesis-naive investigation of the ten genes with strongest statistical evidence for ASD association identified cell proliferation as an additional point of functional convergence (Willsey et al., 2021), consistent with other work in induced pluripotent stem cells (iPSCs) derived from individuals with ASD (Marchetto et al., 2017; Mariani et al., 2015; Adhya et al., 2021). In orthologous work, focusing on the hcASD genes *DYRK1A* and *KATNAL2*, we identified involvement of microtubule dysfunction (Willsey et al., 2018; 2020), raising the possibility that disruption of the mitotic spindle, via alteration of tubulin biology, may explain, at least in part, the proliferation phenotypes among hcASD gene mutations. Consistently, many chromatin regulators, including the ASD-associated genes *ADNP* and *CHD3*, are known to modulate both histones and tubulins (Ostapcuk et al., 2018; Mandel, Rechavi, and Gozes, 2007; Mandel and Gozes, 2007; Sillibourne et al., 2007; Yokoyama et al., 2013; Oz et al., 2014; Bassan et al., 1999; Matsuoka et al., 2008; Park et al., 2016; Koenning et al., 2021; Divinski et al., 2004). These findings suggest that ASD-associated chromatin regulators may play pleiotropic roles, regulating both histones and tubulins, and that disruptions of one, the other, or both may underlie ASD pathobiology.

Thus, we sought to test two hypotheses: first, that ASD-associated genes annotated as chromatin regulators have dual functions in regulating chromatin and tubulin, and, second, that alterations in tubulin-related processes are related to ASD pathobiology in general. To address the first question, we turned to the *in vivo Xenopus* model system and *in vitro* human tissue culture systems. We observed that the five chromatin regulators most strongly associated with ASD (ADNP, CHD8, CHD2, POGZ and KMT5B) (Fu et al., 2022) localize to microtubules both *in vivo* and *in vitro*. Focusing on *CHD2*, we demonstrated that recapitulating ASD-associated haploinsufficiency *in vitro* results in phenotypes consistent with a role in regulating microtubules, including mitotic spindle defects, cell cycle defects, DNA damage and cell death. Moreover, we demonstrated that a missense mutation observed in an individual with ASD disrupts the localization of CHD2 to the mitotic spindle. To address our second hypothesis, namely that derangements in microtubule function contribute to ASD pathobiology writ large, we leveraged the past decade of successful gene discovery in ASD with experimentally derived, tubulin-centric, protein–protein interaction (PPI) data. We determined that hcASD genes are over-represented among tubulin-related proteins and that protein-truncating variants (PTVs) found in people with ASD are more likely to occur within tubulin-related proteins. Overall, these findings strongly suggest that disruption of microtubule biology may be an underappreciated component of ASD pathobiology, underscoring the limitations of relying on annotated function to identify convergent pathological mechanisms, and highlighting the importance of hypothesis-free, functional exploration of risk genes to clarify pleiotropy. Furthermore, as chromatin regulators are implicated in risk for many psychiatric disorders, congenital heart disease and cancer, these results may similarly suggest a broader role for tubulin biology in human disease.

## RESULTS AND DISCUSSION

### ASD-associated chromatin regulators localize to microtubule-based structures

**Fig. 1.**
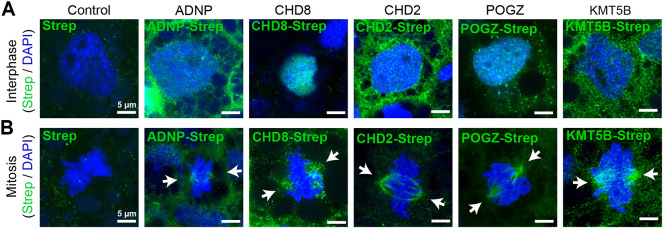
**ASD-associated chromatin regulators localize to microtubules.** (A) Human Strep-tagged constructs for ASD-associated chromatin regulators ADNP, CHD8, CHD2, POGZ and KMT5B (labeled by Strep, green) localize to the nucleus (labeled by DAPI, blue) during interphase when expressed in *Xenopus*. (B) However, these constructs localize to the mitotic spindle during mitosis. Negative controls do not show these localizations. Arrows indicate spindle poles. See also Figs S1-S3.

To test our hypothesis that ASD-associated chromatin regulators also regulate tubulins, we visualized the subcellular localizations of the five chromatin regulators with the strongest statistical evidence for ASD association (ADNP, CHD8, CHD2, POGZ and KMT5B) (Fu et al., 2022). First, we expressed Strep-tagged human cDNAs in *Xenopus* embryos and imaged interphase and mitotic cells, visualizing the Strep-tag and DNA. In interphase cells, ADNP, CHD8, CHD2, POGZ and KMT5B localized to the nucleus, consistent with their reported functions as gene expression regulators (Fig. 1A). However, in metaphase cells, all five proteins localized to microtubule-rich mitotic spindles (Fig. 1B). Negative-control Strep experiments did not show this staining (Fig. 1A,B), nor did a Strep-tagged ETV1 transcription factor, which is not associated with ASD (Fig. S1). We confirmed these localizations *in vitro* in human HEK293T cells (Fig. S2).

As these data depend on overexpression, we also wanted to assay endogenous localization by antibody staining. We validated a Chd2 antibody in *Xenopus* embryos with *chd2* antisense oligonucleotides and western blot analysis of protein lysates. This antibody labeled a single band at the predicted size, which was reduced in injected animals compared with controls (Fig. S3A,B). Next, we stained blastula-stage *Xenopus* embryos, in which it is easy to image mitotic spindles. For all five proteins, we observed localization within the nucleus during interphase and with the spindle during mitosis (Fig. S3C). In human iPSC-derived neural progenitor cells (NPCs), we observed all five proteins in the nucleus during interphase and very diffusely throughout the cytoplasm during mitosis (Fig. S3D). We differentiated NPCs to cortical excitatory neurons and observed both nuclear localization and microtubule localization along axons (Fig. S3E). Together, these data indicate that the five most strongly ASD-associated chromatin regulators can localize to microtubules in multiple cell types across both *in vivo* and *in vitro* models, consistent with potential functional roles in regulating microtubules.

### CHD2 is required for spindle organization, cell cycle progression, genome stability and cell survival

**Fig. 2.**
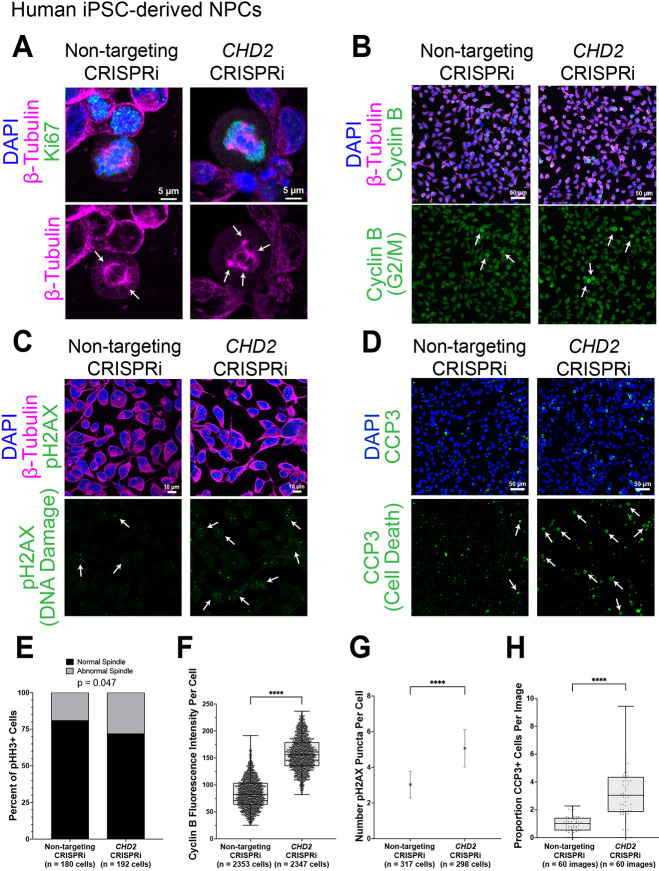
**CHD2 is required for mitotic spindle organization, cell cycle progression, genome stability and cell survival.** (A-D) *CHD2* CRISPRi in human iPSC-derived NPCs causes an increase in mitotic spindle defects (arrows, multipolar spindle) (A), in cyclin B (G2/M marker) fluorescence per cell (B), in pH2AX (DNA damage marker) puncta per nucleus (C), and in the proportion of CCP3 (cell death marker) positive cells (D) compared with non-targeting CRISPRi. (E) Quantification of the data shown in A (χ^2^ test). (F) Quantification of the data shown in B. Box is interquartile range, line is median, and whiskers are maximum to minimum values. (G) Quantification of the data shown in C. Dot is at the mean and lines are 95% confidence intervals. (H) Quantification of the data shown in D. Box is interquartile range, line is median, and whiskers are maximum to minimum values. ****P<0.0001 (non-parametric rank sum test). See also Fig. S4.

Given this evidence that multiple hcASD genes involved in chromatin biology encode proteins that also localize to the spindle, we next sought to interrogate whether ASD-associated mutations alter microtubule biology. As many of these mutations lead to haploinsufficiency, we characterized the impact of *CHD2* loss of function on mitosis, a proliferation-related process that depends on dynamic microtubule regulation. We inhibited *CHD2* using CRISPR interference (CRISPRi) in human iPSC-derived cortical NPCs, an *in vitro* model of a cell type we and others have previously implicated in ASD risk (Willsey et al., 2022; 2021). We confirmed *CHD2* knockdown by qPCR (Fig. S4A) and assayed whether mitotic spindle organization was disrupted. We observed that reduction of *CHD2* led to an increase in the frequency of abnormal mitotic spindles (labeled by β-tubulin) in dividing cells (labeled by phospho-histone H3 staining) compared with non-targeting controls (Fig. 2A,E, Fig. S4B; χ^2^ test *P*=0.047). Defects in spindle organization often lead to cell cycle stalling, DNA damage and cell death; therefore, we assessed each of these phenotypes. *CHD2* reduction caused an increase in cyclin B (Fig. 2B,F; G2/M marker; rank sum test *P*<0.0001) and a decrease in cyclin E (Fig. S4C,D; G1/S marker; rank sum test *P*<0.0001) compared with non-targeting controls, suggesting that a larger proportion of cells were in G2/M phases. This is the expected result if the mitotic spindle checkpoint is activated as a result of abnormal spindle organization and chromosomal mis-segregation, causing cells to stall during M phase (Bharadwaj and Yu, 2004). Abnormal spindle organization also often causes DNA damage, as the spindle cannot properly segregate chromosomes. Indeed, *CHD2* reduction significantly increased the number of phosphorylated H2A histone family member X (pH2AX) puncta per nucleus compared with controls (Fig. 2C,G; rank sum test *P*<0.0001). pH2AX is a canonical marker for improper cell division and resulting chromosomal instability (Rogakou et al., 1998). This result is consistent with a study observing increased DNA damage in iPSC-derived NPCs from people with ASD (Wang et al., 2020). DNA damage can trigger apoptosis, and we also observed an increase in cell death, as measured by cleaved caspase 3 antibody staining (Fig. 2D,H; rank sum test *P*<0.0001). Together, these data point to a role for CHD2 in spindle organization, cell cycle progression, genome stability and cell survival, which could be due to a function in regulating microtubule dynamics.

### A CHD2 missense variant prevents localization to the mitotic spindle

**Fig. 3.**
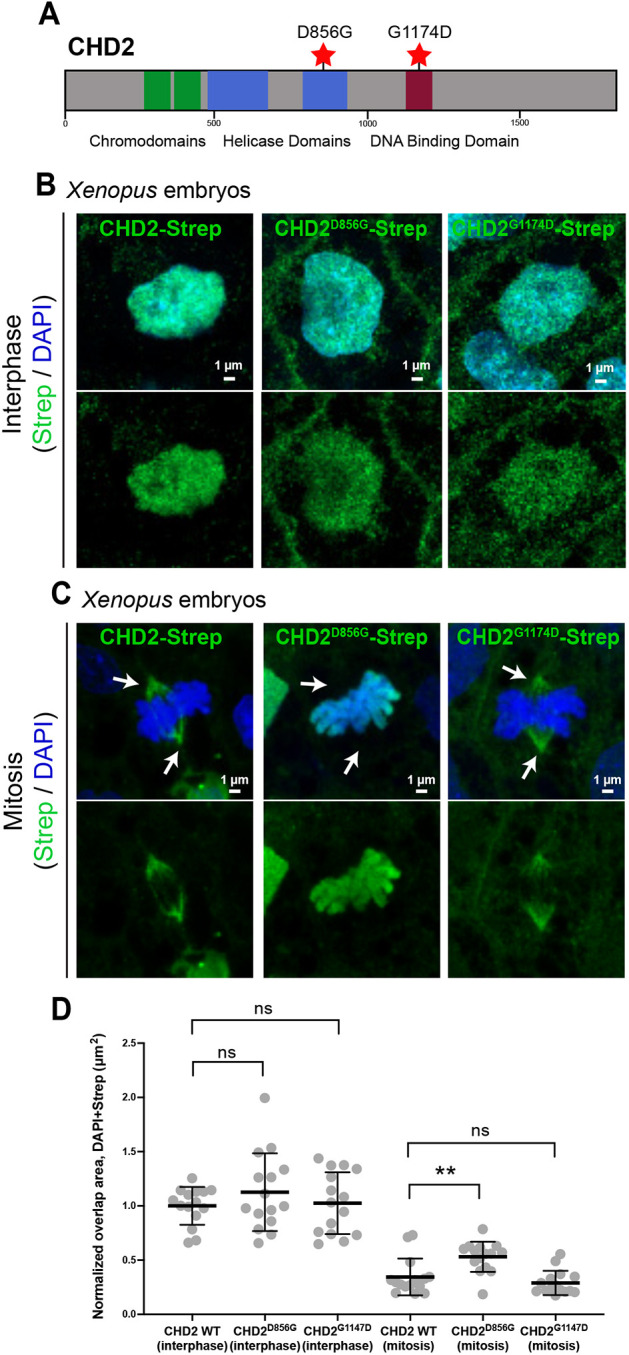
**A missense variant of CHD2 observed in an individual with ASD disrupts spindle localization.** (A) Schematic of human CHD2 protein with functional domains and locations of likely pathogenic missense variants annotated. (B) Strep-tagged human CHD2 and ASD-associated variants CHD2^D856G^ and CHD2^G1174D^ expressed in *Xenopus* localize to the nucleus during interphase. (C) During mitosis, CHD2 and CHD2^G1174D^ localize to spindles during mitosis, whereas CHD2^D856G^ does not and instead remains localized to DNA (DAPI, blue). Arrows indicate spindle poles. (D) Quantification of the area of overlap between Strep and DAPI. Bars are mean and whiskers are interquartile range. **P<0.01 (one-way ANOVA). ns, not significant.

We next reasoned that studying missense variants observed in individuals with ASD might provide additional insights into pathological mechanisms beyond those obtained from a knockdown model. In ASD exome sequencing studies (Satterstrom et al., 2020), three *CHD2* missense variants have been identified. Of these, two (Fig. 3A) have strong evidence for being pathological based on analysis of missense badness, PolyPhen-2, and constraint (MPC) scores (Samocha et al., 2017 preprint). Therefore, we cloned and tagged these two variants, expressed them in *Xenopus*, and localized them. In interphase cells, all constructs localized to the nucleus, coincident with DAPI DNA staining (Fig. 3B,D). However, in mitotic cells, although the reference CHD2 construct and the missense variant CHD2^G1174D^ localized to the spindle, the missense variant CHD2^D856G^ did not, and instead remained associated with DNA throughout mitosis (Fig. 3C,D, Fig. S5). Interestingly, CHD2^D856G^ is within one of the helicase domains, whereas CHD2^G1174D^ is within the DNA-binding domain (Fig. 3A).

### Tubulin-related proteins in general carry risk for autism

**Fig. 4.**
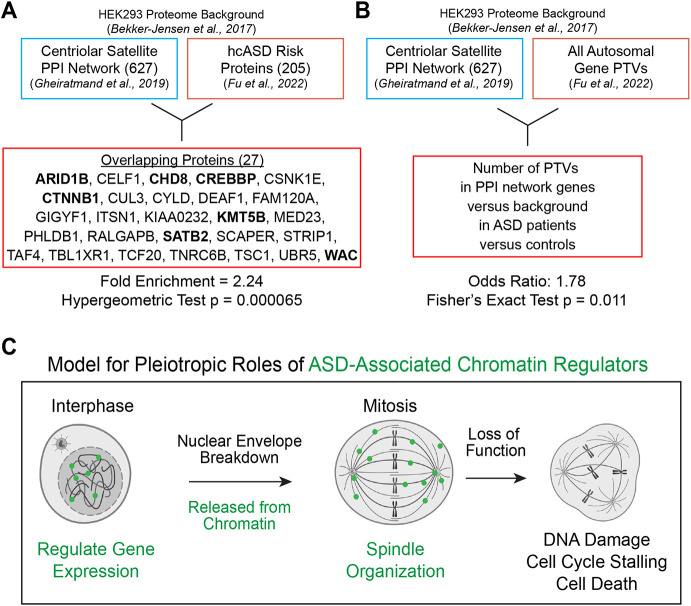
**Enrichment of microtubule-related proteins in ASD.** (A) hcASD risk proteins (taken from Fu et al., 2022) are over-represented in a microtubule-related centriolar satellite proteome (taken from Gheiratmand et al., 2019). A significant number of overlapping proteins (bold) are annotated as chromatin binding. (B) Genes encoding centriolar satellite-associated proteins (Gheiratmand et al., 2019) are more likely to carry protein-truncating variants (PTVs) in individuals with ASD (Fu et al., 2022) compared with non-network genes. (C) Model for pleiotropy of ASD-associated chromatin regulators. During interphase, they localize to nuclei, regulating gene expression. During mitosis, they regulate tubulin and organize the mitotic spindle (inspired by Yokoyama, 2016). In loss of function, the spindle is disorganized, leading to cell cycle defects, DNA damage and death. See also Table S2.

Next, we wanted to test our second hypothesis: namely, that tubulin biology is related to ASD pathobiology generally. Given that centriolar satellites are proposed sites of tubulin post-translational modification (Gheiratmand et al., 2019), we queried a PPI network generated from affinity-purification mass spectrometry of proteins associated with centriolar satellites (Gheiratmand et al., 2019). We tested whether hcASD genes (*n*=205, FDR<0.01; Fu et al., 2022) are over-represented within this network, and observed significant overlap (27/627; fold enrichment 2.24, *P*=0.000066; Fig. 4A, Table S2). Of the 27 overlapping proteins, seven of them (*CHD8*, *KMT5B*, *ARID1B*, *WAC*, *CREBBP*, *CTNNB1*, *SATB2*) are annotated as ‘chromatin binding’ (GO:0003682), an enrichment not expected by chance (odds ratio 4.33, Fisher's exact test *P*=0.0039). We next tested whether these centriolar satellite PPI network interactors were enriched within a large molecular interaction network constructed from hcASD risk proteins and the PCNet (Parsimonious Composite Network) database of molecular interactions (Huang et al., 2018; Willsey et al., 2021). A significant proportion of the centriolar satellite PPI network interactors are present in the ASD interaction network (603/639, fold enrichment 1.12, hypergeometric test *P*=1.99×10^−16^).

Given that the centriolar satellite proteome was generated in HEK293T kidney cells, we next wanted to test whether tubulin-associated proteomes generated in neural cells are also enriched for ASD risk genes. To do this, we used centrosome-associated proteomes generated in human iPSC-derived NPCs and neurons, as centrosomes are microtubule-organizing centers and share many proteins with centriolar satellites (O'Neill et al., 2022). Here, we also observed that hcASD genes (*n*=205, FDR<0.01; Fu et al.) are significantly over-represented in the centrosome-associated networks generated in NPCs (21/698 overlapping, fold enrichment 1.56, *P*=0.027; Table S2) and in neurons (29/727 overlapping, fold enrichment 2.08, *P*=0.00013; Table S2). These results suggest that proteins encoded by ASD risk genes tend to interact with tubulin-associated molecular networks in a variety of cell types, including neurons.

Finally, we investigated whether presence in these tubulin-related PPI networks increases the odds that a gene carries a pathogenic variant in an individual with ASD. Indeed, we observed that genes encoding proteins in the centriolar satellite proteome are significantly more likely to contain PTVs identified in people with ASD, compared with non-network genes and unaffected control individuals (odds ratio 1.78, Fisher's exact test two-tailed *P*=0.011; Fig. 4B, Table S2). However, enrichment of PTVs from individuals with ASD in the other centrosome-associated networks was not statistically significant (Table S2). Thus, consistent with the gene-based tests, these variant-level analyses also implicate tubulin-associated proteins in ASD risk. Additionally, the pattern of enrichment suggests that centriolar satellite proteins may carry particularly strong risk.

In summary, multiple lines of evidence suggest an important role for tubulin biology in ASD pathogenesis. We previously identified a convergent role for ASD risk genes in cell proliferation and implicated microtubule regulation in this process for DYRK1A and KATNAL2 (Willsey et al., 2018; 2020; 2021). Others have described direct microtubule-related functions for ASD risk proteins including chromatin regulators (CHD3 and ADNP), a kinase (DYRK1A) and a microtubule-severing protein (SPAST) (Ori-McKenney et al., 2016; Sillibourne et al., 2007; Oz et al., 2014; Kuo et al., 2019). Here, we have studied five genes selected based on their statistical association with ASD and their annotated function as chromatin regulators (*ADNP*, *CHD8*, *CHD2*, *POGZ*, *KMT5B*), and observe that all five localize to microtubules. We also describe a role for CHD2 in mitosis, with loss of function causing mitotic spindle defects, cell cycle stalling, DNA damage and cell death, all consistent with a role regulating microtubules. We therefore propose a model in which ASD-associated chromatin regulators perform pleiotropic roles (Fig. 4C). Specifically, during interphase, chromatin regulators localize to the nucleus and perform gene expression regulatory functions, but once the nuclear envelope breaks down and the cell enters mitosis, these proteins relocalize to organize the mitotic spindle, as has been proposed for other chromatin modifiers, such as CHD4 (Yokoyama, 2016). Therefore, loss of function leads to mitotic spindle defects, cell cycle stalling, DNA damage and cell death (Fig. 4C).

This model and the results presented here offer potential insight into our prior findings showing that *ADNP*, *CHD8*, *CHD2* or *POGZ* mutant *X. tropicalis* have an apparent expansion of proliferative neural progenitors, but ultimately a smaller brain size (Willsey et al., 2021). In consideration of our new work, our model suggests that the apparent expansion of proliferative cells could be the result of cell cycle stalling during mitosis and that the smaller brain was due to subsequent cell death and/or perturbed proliferation. Consistent with this, individuals with variants in the five genes studied here (*ADNP*, *CHD8*, *CHD2*, *POGZ* or *KMT5B*) commonly present with growth abnormalities and brain size changes (Firth et al., 2009; Bernier et al., 2014; Suls et al., 2013; Assia Batzir et al., 2020; Kawarai et al., 2018; Chen et al., 2022; Eliyahu et al., 2022).

In general, the path from a growing list of large-effect mutations to an actionable understanding of pathobiology has been complicated by, among other things, the extensive pleiotropy of ASD risk genes (Willsey et al., 2021; Sestan and State, 2018; Panagiotakos and Pasca, 2022; Willsey et al., 2022). As a case in point, from the earliest successes in rare variant gene discovery in ASD, genes annotated as chromatin regulators have been over-represented among high confidence genes, leading to the conventional wisdom that pathobiology is due to dysregulation of gene expression (Valencia and Paşca, 2022). For example, the ASD-associated chromatin regulator CHD8 has been extensively studied with respect to its role in chromatin biology. However, mutations appear to have a modest effect on global gene expression (Cotney et al., 2015; Sugathan et al., 2014), and transcriptional effects vary widely between studies (Wade et al., 2018), raising the possibility that CHD8 may play additional roles in ASD beyond directly regulating gene expression. The work presented here broadens the likely relevant functions of such well-established chromatin regulators to include tubulin organization and underscores the value of including hypothesis-naive approaches to studying mechanistic convergence among a diverse set of risk genes as an alternative to relying on annotated function to identify pathobiology.

In addition to observing directly the impact of ASD-associated mutations on tubulin biology, we provide strong evidence for the contribution of tubulin biology more broadly to ASD risk by leveraging *in silico* analyses of PPI resources. hcASD genes are enriched in both centrosome-associated and centriolar satellite-associated networks. We also observe significant ASD genetic risk among proteins associated with centriolar satellites. This is particularly interesting because centriolar satellites are required for the formation of tubulin-based structures, such as centrosomes and cilia, and have been hypothesized to be sites of tubulin post-translational modification (Gheiratmand et al., 2019; Tollenaere et al., 2015; Odabasi et al., 2020; Hall et al., 2023). Parsing the relative contributions of spindles, centrosomes, centriolar satellites, and cilia to ASD pathobiology will be important future work.

Key questions also remain regarding the relative contributions of transcriptional versus microtubule dysregulation to ASD pathobiology. Specifically, for these ‘dual-function’ genes it is unclear whether disruptions of chromatin, microtubules or both underlie ASD pathobiology, especially because we cannot exclude the possibility that the microtubule-related defects seen in our *CHD2* loss-of-function experiments are not due to the protein's chromatin function. Similarly, we observe that a likely pathogenic missense variant (CHD2^D856G^) observed in an individual with ASD does not localize to the spindle, but another likely pathogenic missense variant (CHD2^G1174D^) does. This could be for several reasons. First, this variant may localize correctly but function aberrantly at the spindle. Second, the missense mutation may not be pathogenic. Third, this variant may not impact microtubule biology. Additionally, it is unclear whether these missense variants also impact chromatin biology. Future work interrogating such variants will be necessary to disentangle these possibilities and parse how variants in each of these chromatin regulators affect mitosis and other phenotypes mechanistically. Given the precedent for chromatin regulators (including CHD3 and CHD4) to play independent and direct roles at both chromatin and microtubules, it is likely that these functions are separable. However, testing this is made difficult by the precedent for DNA-binding domains and nuclear localization sequences to be required in some cases for microtubule targeting (Haque et al., 2022; Nachury et al., 2001; Gruss et al., 2001), so deletion constructs in these domains may not be able to discriminate between these functions. Rather, these functions could be separated in the *Xenopus* cell-free cytoplasmic egg extract system, where spindles can form in the absence of nuclei (Heald et al., 1996; Gard and Kirschner, 1987).

The tubulin code and associated post-translational modifications are rich and elaborate (Janke and Magiera, 2020). Microtubule stability and function can vary widely depending on the combinations of tubulin isotypes, modifications and the developmental context, underscoring the diverse functions of tubulin in development, ranging from mitosis to ciliogenesis to cell migration to synaptic transmission (Janke and Magiera, 2020; Lasser et al., 2018; Roll-Mecak, 2020). Understanding how ASD risk proteins intersect microtubules in all these contexts will be important, and determining which, if any, are relevant to pathobiology will be crucial. Given the plethora of microtubule-targeting drugs approved by the United States Food and Drug Administration in the field of oncology, identifying tubulin-related ASD mechanisms may offer a particularly tractable path for the development and testing of rational therapeutic strategies.

## MATERIALS AND METHODS

### Human ASD risk gene plasmid construction

ASD risk gene isoforms were selected based on gene expression in the developing cortex and the presence of exons with ASD-associated variants using Clonotator software (www.willseylab.com/resources). Human cDNA was purchased from third-party companies (CHD8 and ADNP from OriGene; CHD2 from GenScript; POGZ from Dharmacon; KMT5B from DNASU Plasmid Repository) and cloned into pCDNA4 using In-Fusion cloning (Berrow et al., 2007) to add a dual Strep-tag at either the N or C terminus (Table S1). A human ETV1 plasmid was a kind gift from John Wallingford (Tu et al., 2018), which we subcloned into pCDNA4 using In-Fusion cloning. Plasmid inserts were sequence-verified via Sanger sequencing. Variants derived from individuals with ASD were created by site-directed mutagenesis using Q5 High Fidelity Master Mix (NEB M0492L), T4 polynucleotide kinase (NEB M0201L) and T4 DNA ligase (NEB M0202L). Variants were selected by presence in the Satterstrom et al. (2020) dataset with an MPC score >2 (Samocha et al., 2017 preprint).

### *Xenopus* care, husbandry and microinjection

*Xenopus tropicalis* or *laevis* adult breeding animals originated in the Khokha lab (Yale School of Medicine; wild-type *Superman* strain), in the National *Xenopus* Resource (RRID:SCR_013731; Pearl et al., 2012; wild-type *Superman* strain) or from Nasco (Fort Atkinson, WI, USA; wild type). Animals were cared for at the Willsey Lab aquatic facility in a recirculating system and used in accordance with an approved UCSF IACUC protocol (AN183565-02B). Wild-type animals of both sexes were used for these experiments. Natural matings and *in vitro* fertilizations were performed, where human chorionic gonadotropin (Sigma-Aldrich) was used for ovulation according to previously published protocols (Sive, Grainger, and Harland, 2007). Embryos were staged according to Faber and Nieuwkoop (2020). Clutch mates or internal ‘within-animal’ tissue control cells were always used as controls. Parker Picospritzer III, Narishige micromanipulators, and Zeiss Stemi 508 microscopes were used to inject 2 nl of injection mix into one blastomere of two-cell-stage embryos. Plasmids were injected at 20 pg per blastomere. All injected reagents are detailed in Table S1. A fluorescent dextran (Thermo Fisher Scientific, D34679) was co-injected to label the injected side of the embryo, and injected animals were sorted according to fluorescence at neurula stages. Any animals with atypical levels of dextran dye, indicating improper injection, were discarded.

### Chd2 antibody validation in *Xenopus*

For Chd2 antibody validation, 2 nl (3.32 pg) of Chd2 antisense oligonucleotide sequence (5′-GGTTTATCCTCATTCCTCATCATTG-3′) was injected into one-cell-stage *Xenopus laevis* embryos, and a standard western blot was performed on stage 22 lysates from these embryos and uninjected control embryos (Bio-Rad minigel with trans-blot turbo transfer). Chd2 antibody (1:1000, Novus Biologicals, NBP2-32563) and β-actin antibody (1:5000, Sigma-Aldrich, A3853) were detected using horseradish peroxidase-conjugated secondary antibodies (Thermo Fisher Scientific, G21234 and G21040) and ECL reaction (Bio-Rad, 1705062).

### *Xenopus* immunostaining, microscopy and image analyses

Whole-mount immunostaining of blastula- and tailbud-stage animals was carried out according to previously published protocols (Willsey et al., 2018, 2020), including the bleaching step (this step was essential for permeabilization to visualize mitotic spindles in the epidermis) with dyes and DAPI (Thermo Fisher Scientific, D3571) added during the secondary antibody incubation. All staining reagents are detailed in Table S1. Samples were mounted on glass slides (within an area enclosed by a ring of vacuum grease) with PBS and coverslipped. Localization images were acquired either on a Leica SP8 laser-scanning confocal microscope or Zeiss LSM980 confocal microscope with a 63× oil objective. Images were acquired as *z*-stacks at system-optimized intervals and processed in Fiji as maximum intensity projections. When fluorescence intensity was compared between samples, imaging acquisition settings were maintained between samples. To quantify the area of overlap between the Strep antibody and DAPI, five interphase and mitotic cells were imaged from three different embryos per condition. The area of overlap was measured using the free-hand tool in Fiji. Differences between CHD2 wild-type and CHD2 mutant conditions were tested for statistical significance using a one-way ANOVA in GraphPad Prism 9.

### HEK293T cell culture, transfection, immunofluorescence and imaging

Human HEK293T cells (HEK293T/17, obtained from and characterized by ATCC) were maintained at 37°C in complete media consisting of DMEM with high glucose GlutaMAX Supplement (Gibco) and 10% fetal bovine serum, and passaged at 20,000 cells/well using 0.25% Trypsin+EDTA (Gibco). Cells were seeded onto a 96-well optically clear plate (Greiner CELLSTAR plates) that was coated overnight with 5 μg/cm^2^ fibronectin (R&D Systems) in PBS or onto glass coverslips in tissue culture plates. Transfections were performed using the PolyJet *in vitro* DNA transfection reagent kit (SignaGen Laboratories). Forty-eight hours after transfection, cells were fixed in 4% paraformaldehyde at room temperature (RT) for 30 min. Cells were permeabilized for 15 min in PBS with 0.125% Triton X-100 (PBST), and blocked for 45 min with 2% bovine serum albumin in PBST (HEK blocking solution) at RT. Cells were incubated in primary antibodies diluted in HEK blocking solution overnight at 4°C, permeabilized for 15 min at RT, and incubated in secondary antibodies with DAPI (Thermo Fisher Scientific, D3571) in HEK blocking solution for 1 h at RT. Antibodies are detailed in Table S1. Cells were imaged on a Leica SP8 laser-scanning confocal microscope with 40× (for culture plates) or 63× (for glass slides) objectives. Images were acquired as *z*-stacks at system-optimized intervals and processed in Fiji as maximum intensity projections.

### Human iPSC-derived NPCs, neurons, immunofluorescence, imaging and analyses

Non-targeting and *CHD2* CRISPRi iPSC-derived NPCs were generated as described previously (Willsey et al., 2021), from a WTC11 dCas9 KRAB iPSC line. *CHD2* knockdown was confirmed by qPCR using primers 5′-CAGAGAGTGAGCCAGAACAAA-3′ and 5′-CCTTCCACTTGATGAGGTACTG-3′ (Fig. S4). NPCs were plated on Matrigel-coated glass coverslips and fixed in 4% paraformaldehyde. Cells were then permeabilized for 10 min in PBS containing 0.2% Triton X-100 (PBT) and blocked in PBS containing 0.1% Triton X-100 with 5% normal goat serum (NPC blocking solution) for 30 min at RT. Cells were incubated for 1 h at RT in primary antibodies (Table S1) diluted in NPC blocking solution, washed in PBT, incubated in secondary antibodies (Table S1) diluted in NPC blocking solution for 1 h at RT, and washed in PBT and then PBS. Coverslips were mounted with DAPI (Thermo Fisher Scientific, D3571) on glass slides before being imaged. Images were acquired using a Leica SP8 laser-scanning confocal microscope with 10× or 63× objectives. Images were acquired as *z*-stacks at system-optimized intervals, and processed into maximum intensity projections in Fiji.

The proportion of dividing cells (phospho-histone H3 positive) with mitotic spindle defects was determined from images of the mitotic spindle (stained by β-tubulin). All phases of mitosis (prometaphase, metaphase, anaphase and telophase) were included. Spindles were considered normal if they were bipolar (two symmetric spindle poles) and abnormal if they were monopolar (one spindle pole), multipolar (more than two spindle poles) or disorganized (asymmetric bipolar) (see Fig. S4B for examples). Difference in spindle defect proportion was tested with a 2×2 contingency table and a χ^2^ test.

pH2AX puncta were quantified using the CellProfiler 4.1.3 Software (McQuin et al., 2018) as previously described (Popova et al., 2021). Briefly, the ‘Speckle Counting’ example pipeline was modified to produce robust identification of puncta. First, DAPI^+^ nuclei were identified using a diameter range of 70-250 pixels, threshold strategy ‘Adaptive’ and threshold method ‘Otsu’. The pH2AX channel was first enhanced and then masked, and puncta were subsequently counted using a diameter range of 7-10 pixels, threshold strategy ‘Adaptive’ and threshold method ‘Otsu’. A parent-child relationship was then assigned to group pH2AX puncta by DAPI^+^ staining, and the CellProfiler output was exported to Microsoft Excel. The number of pH2AX puncta per nucleus was plotted by condition and tested for statistical significance between conditions using an unpaired non-parametric rank sum test in GraphPad Prism 9. Cyclin B and cyclin E mean fluorescence intensity was quantified using the ‘Analyze Particle’ function after thresholding in Fiji and tested for statistical significance between conditions using an unpaired non-parametric rank sum test in GraphPad Prism 9. For CCP3, cells with positive antibody staining were marked and counted manually in Fiji. Differences in the ratio CCP3^+^ cells/total number DAPI^+^ cells between CRISPRi conditions were tested for statistical significance using an unpaired non-parametric rank sum test in GraphPad Prism 9.

NPCs were differentiated into neurons according to a previously described protocol (Willsey et al., 2021), with the exception of the matrix, which was poly-L-ornithine, laminin and fibronectin instead of Matrigel.

### Systems biological analyses

We first created a custom background for hcASD risk gene enrichment by intersecting proteins expressed in HEK293T cells (Bekker-Jensen et al., 2017) with all the autosomal genes queried in a previously published dataset (Fu et al., 2022), resulting in 10,667 unique proteins. In this background, 205 hcASD genes (Fu et al., 2022), 698 centrosomal proteins (NPCs) (O'Neill et al., 2022), 727 centrosomal proteins (neurons) (O'Neill et al., 2022), 590 ‘microtubule-independent’ centrosomal proteins (O'Neill et al., 2022) and 627 centriolar satellite proteins (Gheiratmand et al., 2019) were present (Table S2). We overlapped the PPI network proteins with the hcASD risk genes and tested enrichment by hypergeometric test.

Next, we assessed whether the 27 hcASD/centriolar satellite genes are enriched for genes that are annotated as ‘chromatin binding’ in gene ontology (GO:0003682). We defined the universe of genes to be the union of *n*=205 hcASD genes and *n*=627 centriolar satellite genes (total *n*=805 genes, which includes *n*=65 of *n*=572 chromatin binding genes in GO:0003682). We conducted a Fisher's exact test using a 2×2 contingency table using the variables hcASD+centriolar satellite gene (yes/no) and chromatin binding (yes/no).

Next, we tested whether an interaction network built from PCNet (Huang et al., 2018) around hcASD risk genes (Willsey et al., 2021) is enriched for these centriolar satellite protein interactors. To do this, we created a custom background by intersecting proteins from PCNet with all autosomal genes queried in ASD exome sequencing studies (Fu et al., 2022) and proteins expressed in HEK293 T cell lines (Bekker-Jensen et al., 2017). Using this background, 639 of the centriolar satellite interactors remained; 603 of these proteins were present in the hcASD network built from PCNet, and this enrichment was tested by hypergeometric test.

Finally, we performed a Fisher's exact test to calculate the significance and odds ratio for *de novo* PTVs (Fu et al., 2022) occurring in centrosome or centriolar satellite PPI genes (O'Neill et al., 2022; Gheiratmand et al., 2019) (Table S2) versus non-network genes in individuals with ASD versus unaffected control individuals.

## Supplementary Material

10.1242/develop.201515_sup1 Supplementary information
