# Supplementary material for: Pleiotropy of autism-associated chromatin regulators
Source: Development. 2023 Jul 18;150(14):dev201515. doi: 10.1242/dev.201515 (PMC10399978; doi:10.1242/dev.201515)
Supplement: Supplementary information [file develop-150-201515-s1.pdf]

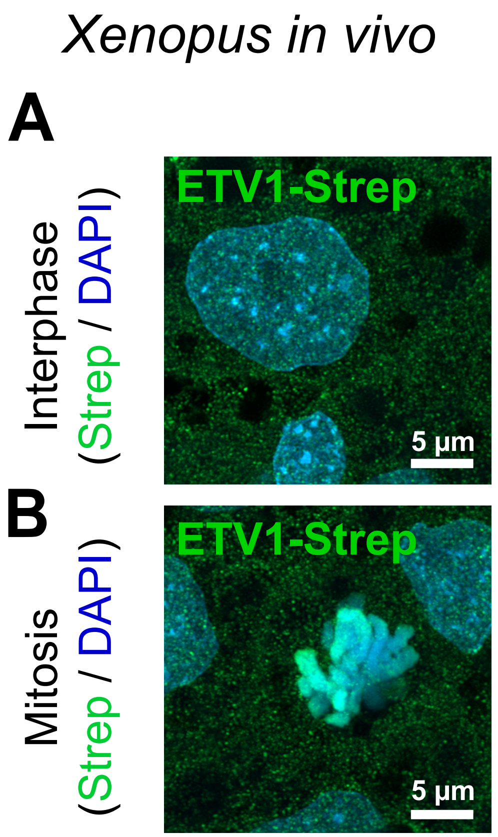

Fig.S1. Control plasmid ETV1-Strep does not localize to the mitotic spindle. Strep-tagged ETV1 transcription factor, which is not associated with ASD, does not localize to the mitotic spindle during mitosis. Related to Figure 1.

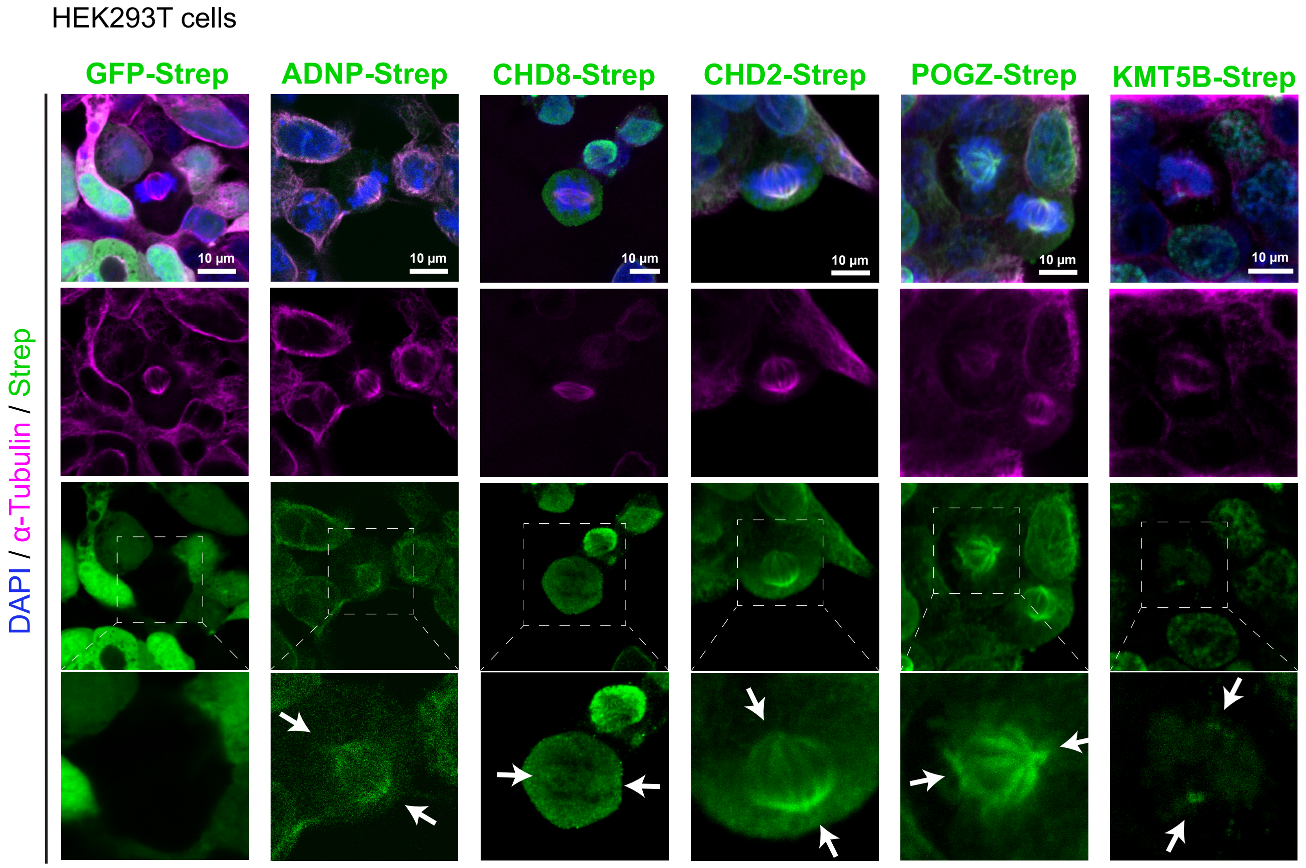

Fig. S2. ASD-associated chromatin regulators localize to microtubules *in vitro*. ASD-associated chromatin regulators (ADNP, CHD8, CHD2, POGZ, and KMT5B) localize to the mitotic spindle or centrosome when expressed in HEK293T cells, while a control GFP-Strep construct does not. Bottom panel is a higher-magnification view of the boxed area in the panel above. Arrows point to the spindle poles. Related to Figure 1.

Development • Supplementary information

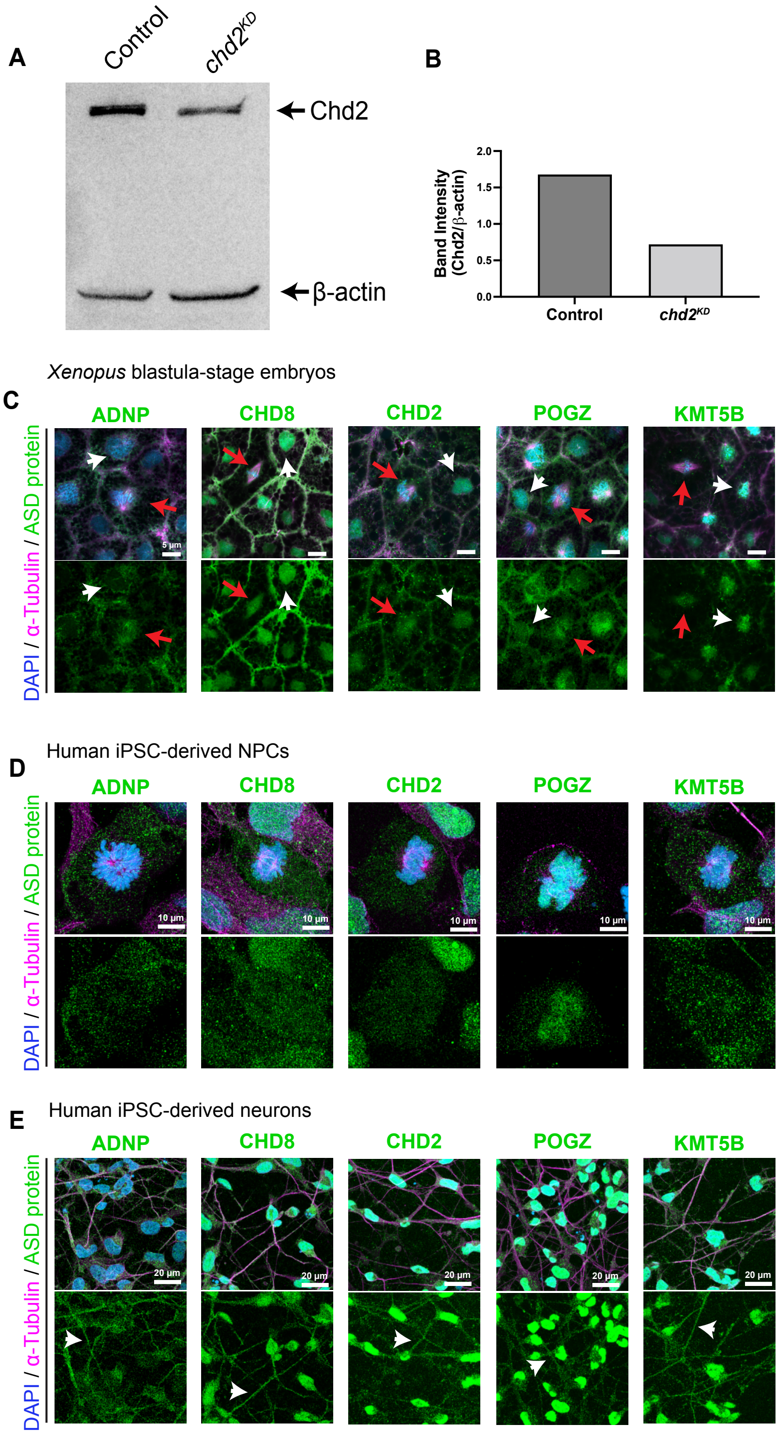

Fig. S3. Antibody stainings for ASD-associated chromatin regulators. (A) Chd2 antibody validation by western blot of control embryos and embryos injected with antisense oligonucle-otides targeting *chd2*. (B) Chd2 knock-down (*chd2KD*) causes a reduction in Chd2 protein, relative to beta-Actin. (C) Staining of *X. laevis* blastula-stage embryos with ASD protein antibodies. White arrows indicate interphase cells, while red arrows indicate mitotic cells. (D) Human iPSC-derived cortical NPCs stained with the ASD protein antibodies. (E) Human iPSC-derived cortical neurons stained with the ASD protein antibodies. White arrows indicate axonal staining. Related to Figure 1.

Development • Supplementary information

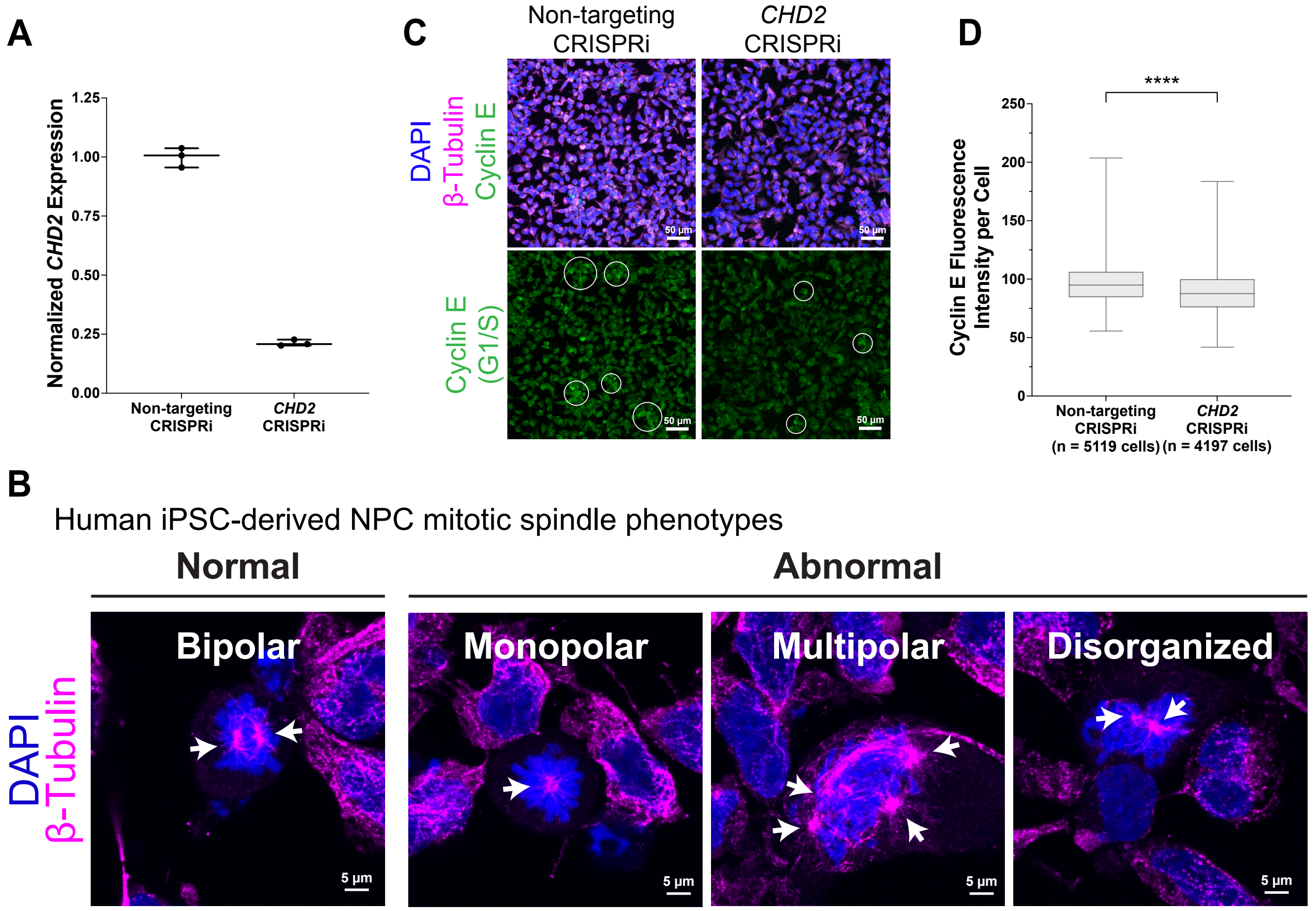

Fig. S4. *CHD2* knockdown validation, spindle defect examples, and cyclin E staining. (A) *CHD2* expression is reduced following CRISPRi targeting of *CHD2*, compared to a non-target-ing control CRISPRi line, as determined by qPCR. (B) Examples of normal and abnormal mitotic spindles for Figure 2A. (C) CRISPRi of *CHD2* causes a significant decrease in cyclin E (G2/M marker) fluorescence per cell compared to a non-targeting CRISPRi line. (D) Quantification of C. Box is 25-75% interquartile range, line is median, and whiskers are max to min. **** represents $p < 0.0001$ by rank sum test. Related to Figure 2.

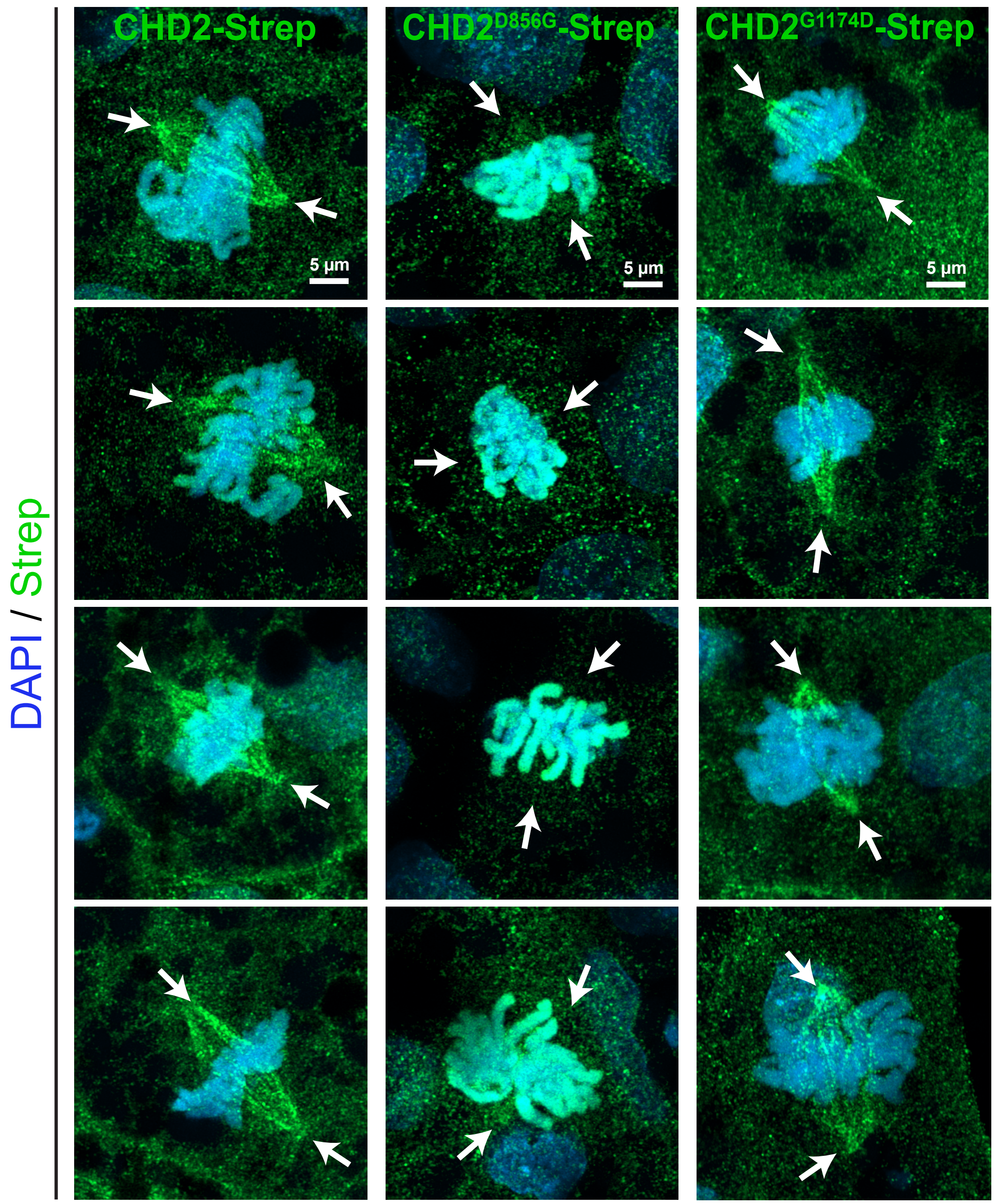

Fig. S5. Example images of CHD2 mutant localizations in *Xenopus*. Additional images of Strep-tagged CHD2 construct localizations in mitotic cells, when expressed in *Xenopus*. CHD2 and mutant CHD2-G1174D localize to the mitotic spindle, while CHD2-D856G remains associated with DNA. Arrows indicate the spindle poles. Related to Figure 3.

Table S1. Reagents used in this work. The first tab lists infomation for the plasmids generated for human hcASD risk gene localizations in *Xenopus*. The second tab contains all the information on antibodies used in this study.

Click here to download Table S1

Table S2. Results of systems analyses. The first tab lists the full lists of hcASD risk genes and proteome components tested for overlap and enrichment. The second tab lists the genes that overlap between these lists. The third tab has the enrichment results from these overlapping gene sets. The fourth tab has the results from the ASD genetic risk tests.

Click here to download Table S2
