# Supplementary material for: Pleiotropy of autism-associated chromatin regulators
Source: Development. 2023 Jul 18;150(14):dev201515. doi: 10.1242/dev.201515 (PMC10399978; doi:10.1242/dev.201515)
Supplement: Supplementary file 1 [file develop-150-201515_review_history.pdf]

Pleiotropy of autism-associated chromatin regulators

Micaela Lasser, Nawei Sun, Yuxiao Xu, Sheng Wang, Sam Drake, Karen Law, Silvano Gonzalez, Belinda Wang, Vanessa Drury, Octavio Castillo, Yefim Zaltsman, Jeanselle Dea, Ethel Bader, Kate E. McCluskey, Matthew W. State, Arthur Jeremy Willsey and Helen Rankin Willsey
DOI: 10.1242/dev.201515

Editor: Francois Guillemot

Review timeline

Original submission:	7 December 2022
Editorial decision:	26 January 2023
First revision received:	25 May 2023
Editorial decision:	9 June 2023
Second revision received:	14 June 2023
Accepted:	19 June 2023

Original submission

First decision letter

MS ID#: DEVELOP/2022/201515

MS TITLE: Pleiotropy of autism-associated chromatin regulators

AUTHORS: Micaela Lasser, Nawei Sun, Yuxiao Xu, Karen Law, Silvano Gonzalez, Belinda Wang, Vanessa Drury, Sam Drake, Yefim Zaltsman, Jeanselle Dea, Ethel Bader, Kate E. McCluskey, Matthew W. State, Arthur Jeremy Willsey, and Helen Rankin Willsey

I have now received the reports of three referees on your manuscript and I have reached a decision. The referees' comments are appended below, or you can access them online: please go to BenchPress and click on the 'Manuscripts with Decisions' queue in the Author Area.

As you will see, all the referees express great interest in your work, but they also have significant criticisms and recommend a substantial revision of your manuscript before we can consider publication. In particular, referee1 asks that you examine the spindle association of some of the endogenous chromatin proteins, and both referees 1 and 3 request that you investigate further CHD2 function and its relation to spindle binding versus DNA binding, e.g. by examining the functions of the CHD2 D856G and CHD2 G1174D mutant forms. If you are able to revise the manuscript along the lines suggested, which may involve further experiments, I will be happy to receive a revised version of the manuscript. Your revised paper will be re-reviewed by the original referees, and its acceptance will depend on your addressing satisfactorily all their major concerns. Please also note that Development will normally permit only one round of major revision.If it would be helpful, you are welcome to contact us to discuss your revision in greater detail. Please send us a point-by-point response indicating your plans for addressing the refereeâ€™s comments, and we will look over this and provide further guidance.

Please attend to all of the reviewers' comments and ensure that you clearly highlight all changes made in the revised manuscript. Please avoid using 'Tracked changes' in Word files as these are lost in PDF conversion. I should be grateful if you would also provide a point-by-point response detailing

© 2023. Published by The Company of Biologists under the terms of the Creative Commons Attribution License (https://creativecommons.org/licenses/by/4.0/).

how you have dealt with the points raised by the reviewers in the 'Response to Reviewers' box. If you do not agree with any of their criticisms or suggestions please explain clearly why this is so.

<u>Reviewer 1</u>

Advance summary and potential significance to field

Here the authors demonstrate that 5 chromatin factors whose mutations has been associated with autism spectrum disorders (ASD) are located at the spindle apparatus during M-phase when expressed in a tagged form in xenopus. It would be important, however, to show this also for endogenous proteins, ideally in the hIPSC-derived neural stem cells. The authors further show that knock-down of Chd2 results in spindle aberrations, increase in gH2AX foci and activated Caspase 3+ human iPSC-derived neural stem cells. As knock-down cannot discriminate between the chromatin versus spindle function, it would be important to demonstrate an effect due to spindle rather than chromatin functions. This may be possible with the mutants of Chd2 that the authors examine next, showing that Chd2-D856G fails to localize to the spindle while Chd2G1174D localized to the spindle. Could the authors check for chromatin functions of these mutations to then explore the effects of the former versus the later mutation on cell cycle progression and aberrant spindle formation? Rather than moving into the mechanism of Chd2 the authors chose to zoom out and overlay centrosomal satellite protein interactors with ASD risk proteins reporting a significant enrichment. Why did the authors choose the overlay with the centrosome satellite proteins? Indeed, such an enrichment of ASD gene variants has been found for the entire centrosome proteome by OÂ'Neill et al., Science 2022. Likewise, the authors find significantly enriched likelihood in protein truncating variants identified in ASD patients amongst the satellite proteome. This is an interesting manuscript as it proposes a key role of spindle formation in ASD pathology including the mutations in alleged chromatin interactors. However, the prove of this point for at least one gene variant is still missing.

Suggestions:
1) Please provide immunostainings for some of the proteins, e.g. Chd2, at endogenous levels in human iPSC-derived NSCs.
2) Please provide quantifications and statistics for the localization of Chd2 mutant proteins shown in Figure 3 and the 5 proteins shown in Figure 1.
3) It would be important to show for one protein, probably Chd2, that indeed loss of its spindle localization (with intact chromatin function) contributes to a phenotype. It could be interesting to generate iPSCs with the 2 Chd2 mutations for this purpose, or express the variants with spindle localization or not in exogenous cells and explore the ensuing phenotype.
4) Regarding the last part with the overlay of ASD mutations and the centrosome satellite proteome the authors should explain why they decided on this proteome, rather than any other centrosome proteome. It may be interesting to overlay this also for other parts of the centrosome, e.g. the interactome of distal and subdistal appendages as published by OÂ'neill et al. 2022

Comments for the author

Suggestions:
1) Please provide immunostainings for some of the proteins, e.g. Chd2, at endogenous levels in human iPSC-derived NSCs.
2) Please provide quantifications and statistics for the localization of Chd2 mutant proteins shown in Figure 3 and the 5 proteins shown in Figure 1.
3) It would be important to show for one protein, probably Chd2, that indeed loss of its spindle localization (with intact chromatin function) contributes to a phenotype. It could be interesting to generate iPSCs with the 2 Chd2 mutations for this purpose, or express the variants with spindle localization or not in exogenous cells and explore the ensuing phenotype.
4) Regarding the last part with the overlay of ASD mutations and the centrosome satellite proteome the authors should explain why they decided on this proteome, rather than any other centrosome proteome. It may be interesting to overlay this also for other parts of the centrosome, e.g. the interactome of distal and subdistal appendages as published by O'neill et al. 2022

© 2023. Published by The Company of Biologists under the terms of the Creative Commons Attribution License (https://creativecommons.org/licenses/by/4.0/).

Reviewer 2

Advance summary and potential significance to field

This interesting short paper by Lasser et al introduces a new hypothesis for protein function of proteins encoded by high confidence autism spectrum disorder (hcASD) risk genes. Many of these genes encode proteins with well-documented functions as chromatin regulators that bind histones. Here, the authors propose that these proteins actually have pleiotropic effects arising from their association with and regulation of the mitotic spindle. The authors show that several such proteins localize to the spindle in metaphase cells, and show that knockdown of one such protein, CHD2, results in abnormal mitotic spindles, cell cycle stalling, and DNA damage. They go on to show a statistical enrichment for autism risk among microtubule associated proteins in general. All together, this paper presents a new and interesting observation, with an equally interesting hypothesis, and provides a useful cautionary tale for too-narrowly interpreting protein function or localization annotations in Gene Ontology analysis. However, some additional controls and fleshing out of mechanism would be necessary before publication in Development.

Comments for the author

Major comments:
1. Although the authors include Strep-alone as a control for Figure 1, I think it would be important to show that not all CHD or TFs in these families localize to the spindle. This would rule out any unexpected artifacts of strep tagging or other aspects of the staining protocol. I think this could be fairly simple: just choose a couple closely related proteins to those shown in Figure 1 that are not associated with ASD, and show that they do not localize to the spindle when similarly tagged with Strep.

2. My principal concern with the paper in its current form is that it's not at all clear what the association of each protein with the spindle does. For example, it could be that the proteins are required for initial assembly of the spindle, or for MTs to associate with kinetochores, or for maintenance of spindle polarity, etc, etc. Along the same lines, it's hard to rule out that some defects in mitosis might not derive from DNA-associated functions of these proteins, since if DNA is not properly tightly packaged into prophase chromosomes one would also expect defects in cell cycle progression and DNA damage. I think this ambiguity could be cleared up considerably if the authors undertook a basic structure/function analysis of CHD2, in which they make deletion constructs for the chromodomains, helicase domains, and DNA binding domains, and ask which of these still result in localization to MTs. A more rigorous analysis would also test the functionality of each construct.

3. A more minor point, but still on this theme, is that I think the authors' speculation that CHD2 functional helicase activity but not DNA binding is required for spindle localization (lines 163-164) is premature at this stage. Right now all we know is that one point mutation changes localization and the other does not. But a structure function analysis of CHD2 as proposed above would also address this concern.

Reviewer 3

Advance summary and potential significance to field

These authors had previouslyidentified evidence for the involvement of microtubule dysfunction in ASD. In this work they first show that five chromatin regulators most strongly associated with ASD (ADNP CHD8, CHD2, POGZ, and SUV420H1/KMT5B) might play a role in microtubule biology. They found that all five localized to microtubules of the mitotic spindle in in both Xenopus embryos and in human HEK293 cells. They next asked if CHD2 loss-of-function would lead to defects in mitosis. They inhibited CHD2 expression via in human induced pluripotent stem cell (iPSC) derived cortical neural progenitor cells using CRISPRi and observed a modest increase in abnormal mitotic spindles. They also found that CHD2 reduction led to increased Cyclin B and decreased Cyclin E consistent with activation of a mitotic spindle checkpoint. They also observed an increase in pH2AX puncta,

© 2023. Published by The Company of Biologists under the terms of the Creative Commons Attribution License (https://creativecommons.org/licenses/by/4.0/).

consistent with DNA damage, and an increase in cell death. Finally they generated ASD-associated missense mutations in CHD2, expressed them in Xenopus and found that one of these, CHD2D856G failed to localize to the mitotic spindle. Interestingly this mutation lies within the helicase domain. Overall this is a brief but interesting research report. It would be appropriate for publication in development following revisions to address a few points.

Comments for the author

1) The authors should further address whether CHD2 localizes to mitotic spindles across stages and cell types. They look at HEK293 cells and also epidermis of Xenopus tailbud stage embryos. The latter seeming an odd choice - if one was going to look at epidermis one could do so at earlier stages. Does CHD2 localize to the spindle of early embryonic blastomeres and does it in neural progenitor cells as later stages?
2) in examining the localization of the ASD-associated mutations in Figure 3 the authors should also show images for localization in interphase cells. There seems to be a significant increase in association with chromatin for CHD2D856G. It would be good to show more than a single nucleus, show that the three CHD2 proteins are being expressed at equivalent levels, and quantify localization differences.
3)It is surprising that loss of function data is only provided for the iPSC-derived cortical neural progenitors not the embryos. These authors have previously published that CRiSPR-mediated knock down of CHD2 leads to decreased telencehphalon size. The authors should attempt to rescue this phenotype with wt CHD2 vs CHD2D856G vs CHD2G1174D. This would provide important information on whether is is the DNA binding domain, the helicase domain (and by proxy spindle binding) or both that contribute to the telencephalon phenotype.

First revision

<u>Author response to reviewers' comments</u>

Dear Reviewers,

Thank you for your timely and thoughtful feedback. See below in blue for our responses to your comments. We appreciate your help improving the manuscript.

Best,
Helen

Reviewer 1
Advance Summary and Potential Significance to Field:
Here the authors demonstrate that 5 chromatin factors whose mutations has been associated with autism spectrum disorders (ASD) are located at the spindle apparatus during M-phase when expressed in a tagged form in xenopus. It would be important, however, to show this also for endogenous proteins, ideally in the hIPSC-derived neural stem cells.

We appreciate the need for endogenous localizations to support our overexpression work. In the revised manuscript we now show endogenous localizations *in vivo* for all 5 chromatin factors on mitotic spindles in early *Xenopus* embryonic blastula cells, where the cells and spindle are exceptionally large, which aids detection of endogenous proteins (Figure S3C). We also validate the CHD2 antibody by western blotting and antisense oligonucleotides (Figure S3A-B). We also include these antibody stainings for all 5 proteins in human iPSC-derived neural progenitor cells (NPCs; Figure S3D) and human iPSC-derived neurons (Figure S3E). In NPCs, we observe nuclear localization during interphase (as expected), and during M phase the signal is very diffuse, but there is overlap with the microtubules of the spindle. In neurons, we observe localization within the nucleus (as expected), as well as along axonal microtubules.

We also would like to highlight that generation of the mass spectrometry-based protein-protein interaction (PPI) network analyzed in Figure 4 did not rely on overexpression of ASD-associated

© 2023. Published by The Company of Biologists under the terms of the Creative Commons Attribution License (https://creativecommons.org/licenses/by/4.0/).

‘chromatin’ proteins in HEK293T cells (i.e. the endogenous protein was detected). Regardless, in the revised manuscript, we now expand these analyses to include the centrosomal PPI data from O’Neill et al (Science 2022; PMID: 35709258), generated from human iPSC-derived NPCs and neurons. Again, we detect significant enrichment of ASD-associated proteins within these networks (Table S2). Altogether, there is compelling evidence that ASD-associated proteins, including those annotated as ‘chromatin regulators,’ localize to microtubule-rich structures endogenously.

The authors further show that knock-down of Chd2 results in spindle aberrations, increase in gH2AX foci and activated Caspase 3+ human iPSC-derived neural stem cells. As knock-down cannot discriminate between the chromatin versus spindle function, it would be important to demonstrate an effect due to spindle rather than chromatin functions. This may be possible with the mutants of Chd2 that the authors examine next, showing that Chd2-D856G fails to localize to the spindle while Chd2G1174D localized to the spindle. Could the authors check for chromatin functions of these mutations to then explore the effects of the former versus the later mutation on cell cycle progression and aberrant spindle formation?

We agree with the reviewer that it would be compelling to separate the nuclear and cytoplasmic functions of these proteins cleanly. The reviewer is correct that we show that CHD2-G1174D localizes to the spindle, while CHD2-D856G does not. However, it is possible that the mutant that localizes properly still cannot execute its proper function once there, and therefore still represents a spindle defect mutant (rather than a clean chromatin-only effect). This is addressed in the discussion.

Going forward, it will be difficult to separate these functions in cells. One could imagine deleting the DNA-binding domain and assessing for defects that would only act through chromatin-related functions. However, DNA-binding domains are known to target transcription factors (like GLI) to microtubules (Haque et al 2022, PMID: 35725768). Similarly, importins, Ran, and associated nuclear-localization sequences (NLSs) are involved in mitotic spindle formation (Natchury et al., Cell 2001; PMID: 11163243). Indeed, NLSs are known to sequester spindle assembly factors in the nucleus during interphase to avoid premature spindle formation (a nice summary is in the introduction of Ciciarello et al., 2004; PMID: 15572412). There appears to be an intricate link between nuclear and spindle targeting, and mutants or deletion constructs may not be able to discriminate between these functions. Similarly, a NLS mutant, which is constitutively localized to the cytoplasm, may represent a gain-of-function spindle phenotype, which again, would be difficult to interpret with respect to separating chromatin and spindle functions. For these reasons, we chose a clean loss-of-function approach first with the CRISPRi, to model the haploinsufficiency seen in patients to query whether spindle-related phenotypes were present at all, which they are. In future work, we plan to use the *Xenopus* egg extract system (where spindles can form in the absence of nuclei and transcription) to be able to separate these functions, but believe this work is beyond the scope of this manuscript.

Rather than moving into the mechanism of Chd2 the authors chose to zoom out and overlay centrosomal satellite protein interactors with ASD risk proteins reporting a significant enrichment. Why did the authors choose the overlay with the centrosome satellite proteins? Indeed, such an enrichment of ASD gene variants has been found for the entire centrosome proteome by O’Neill et al., Science 2022. Likewise, the authors find significantly enriched likelihood in protein truncating variants identified in ASD patients amongst the satellite proteome.

We originally chose to test overrepresentation among the centriolar satellite proteome since that is a proposed location for tubulin modification by chromatin modifiers (Ghieratmand et al; PMID: 31304627). Nevertheless, we thank the reviewer for pointing us to O’Neill et al (2022), especially as it provides the opportunity to replicate our findings in tubulin-related proteomes from NPCs and neurons. As with the centriolar satellite proteome, we observe significant overrepresentation of hcASD risk genes within the NPC and neuron centrosomal proteomes from O’Neill et al (Table S2). We have included these results in the main text.

This is an interesting manuscript as it proposes a key role of spindle formation in ASD pathology including the mutations in alleged chromatin interactors. However, the prove of this point for at least one gene variant is still missing.

© 2023. Published by The Company of Biologists under the terms of the Creative Commons Attribution License (https://creativecommons.org/licenses/by/4.0/).

CHD2 likely-gene-disrupting variants have been described in ASD (Fu et al 2022; PMID: 35982160); therefore we modeled CHD2 loss-of-function using CRISPRi (Figure 2). In this case, we observe spindle defects, cell cycle defects, and cell death. Therefore, we have shown that an annotated 'chromatin modifier' is required for proper mitotic spindle formation. We agree that there is much more to do in terms of mechanistic detail and potential differences between specific missense variants, and hope that this manuscript spurs others to consider a possible role for ASD risk proteins, including those classically annotated at chromatin, in microtubule-related biology.

Comments for the Author:

Suggestions:
1) Please provide immunostainings for some of the proteins, e.g. Chd2, at endogenous levels in human iPSC-derived NSCs.

We thank the reviewer for this suggestion. We now include antibody staining for all 5 of the proteins in early *Xenopus* embryos (where the cells are extraordinarily large and easy to image spindles) (Figure S3C), iPSC-derived NPCs (Figure S3D), and iPSC-derived neurons (Figure S3E). Please see comment above for more detail.

2) Please provide quantifications and statistics for the localization of Chd2 mutant proteins shown in Figure 3 and the 5 proteins shown in Figure 1.

We now include quantifications and statistics for the Chd2 mutant protein localizations in Figure 3D. We also include a supplemental figure with additional images (Figure S5). For Figure 1, we observed the spindle localization in every mitotic cell we assayed that received the construct. We deduce that the reviewer is asking for a quantification to ensure that these results are robust, and so now in the revised manuscript we include endogenous localizations (Figure S3), an additional Strep-tagged control transcription factor (Figure S1), and expand our analysis of mass spectrometry-derived protein-protein interactions (Figure 4, Table S2). We also validated the Chd2 antibody *in vivo* (Figure S3A-B).

3) It would be important to show for one protein, probably Chd2, that indeed loss of its spindle localization (with intact chromatin function) contributes to a phenotype. It could be interesting to generate iPSCs with the 2 Chd2 mutations for this purpose, or express the variants with spindle localization or not in exogenous cells and explore the ensuing phenotype.

We agree that it would be ideal to be able to separate nuclear and cytoplasmic functions for these chromatin regulators, but, as discussed above, we think separating them is very difficult since the same domains (eg, DNA-binding domains) can function in both nuclear and spindle localization. While these mutants show differential localization, they may not differ in their functionality. We did try and express these constructs in HEK293T cells and in *Xenopus*, but did not observe any significant phenotypes with respect to DNA damage or cell death on their own.

4) Regarding the last part with the overlay of ASD mutations and the centrosome satellite proteome the authors should explain why they decided on this proteome, rather than any other centrosome proteome. It may be interesting to overlay this also for other parts of the centrosome, e.g. the interactome of distal and subdistal appendages as published by O'Neill et al. 2022

We thank the reviewer for this suggestion and refer you to our explanation of our revised work within your broader comments above. Briefly, we now provide rationale for querying the centriolar satellite proteome, compare our results to those in O'Neill et al., and comment on the differences between satellites and centrosomes. Indeed, it seems the autism-associated chromatin modifiers are mostly found in the centriolar satellites, compared to the centrosomes (Table S2).

Reviewer 2
Advance Summary and Potential Significance to Field:
This interesting short paper by Lasser et al introduces a new hypothesis for protein function of proteins encoded by high confidence autism spectrum disorder (hcASD) risk genes. Many of these

© 2023. Published by The Company of Biologists under the terms of the Creative Commons Attribution License (https://creativecommons.org/licenses/by/4.0/).

genes encode proteins with well-documented functions as chromatin regulators that bind histones. Here, the authors propose that these proteins actually have pleiotropic effects arising from their association with and regulation of the mitotic spindle. The authors show that several such proteins localize to the spindle in metaphase cells, and show that knockdown of one such protein, CHD2, results in abnormal mitotic spindles, cell cycle stalling, and DNA damage. They go on to show a statistical enrichment for autism risk among microtubule associated proteins in general. All together, this paper presents a new and interesting observation, with an equally interesting hypothesis, and provides a useful cautionary tale for too-narrowly interpreting protein function or localization annotations in Gene Ontology analysis. However, some additional controls and fleshing out of mechanism would be necessary before publication in Development.

We appreciate the reviewer's comments and positive outlook on publication. We have addressed your comments below.

Reviewer 2 Comments for the Author:
Major comments:

1. Although the authors include Strep-alone as a control for Figure 1, I think it would be important to show that not all CHD or TFs in these families localize to the spindle. This would rule out any unexpected artifacts of strep tagging or other aspects of the staining protocol. I think this could be fairly simple: just choose a couple closely related proteins to those shown in Figure 1 that are not associated with ASD, and show that they do not localize to the spindle when similarly tagged with Strep.

We appreciate this comment and the request for more controls for the localizations in Figure 1. We have included multiple new experiments in the revised manuscript to address this point. First, we now include endogenous localizations of all 5 proteins in multiple cell types with experiments that do not rely on over-expression or the strep antibody (Figure S3). We also validate the CHD2 antibody used by western blotting and antisense oligonucleotides (Figure S3A-B). Similarly, the mass spectrometry protein-protein interaction results in Figure 4 also do not depend on the strep antibody or over-expression of the ASD proteins, and there we see significant association of these chromatin regulators with centriolar satellite proteins. In the revised manuscript, we have expanded these analyses to a larger ASD sequencing dataset and additional mass-spectrometry datasets, and our results hold (Figure 4, Table S2).

Nevertheless, we appreciate the concern with strep-tagging, so we inserted the human sequence for transcription factor ETV1 into our strep vector and localized it in interphase and metaphase cells in *Xenopus*. Here we observed nuclear localization during interphase as expected, and did not observe spindle localization during metaphase (Figure S1). We chose this transcription factor for several reasons. First, spindle localizations have been published previously for CHD3 and CHD4, so we think that spindle localization may indeed be a broader feature of CHDs. We did not test an additional CHD that doesn't have evidence for ASD risk, because the way that ASD risk is assigned is incomplete- lethal variants, for example, would not be identified in human exome sequencing. Therefore, absence on an ASD risk gene list does not mean the gene does not carry risk. For this reason we were hesitant to interpret results from other gene family members. That being said, ETV1 is not known to carry ASD risk (Fu et el, FDR = 0.80) and has been shown to have nuclear localization when expressed in *Xenopus* (Tu et al, 2018; PMID: 29180514), so we subcloned it into our Strep-tag vector and tried it and did not observe spindle localization. Together, these new experiments provide additional evidence that these ASD-associated chromatin factors are associated with microtubules, in addition to their canonical nuclear localizations.

2. My principal concern with the paper in its current form is that it's not at all clear what the association of each protein with the spindle does. For example, it could be that the proteins are required for initial assembly of the spindle, or for MTs to associate with kinetochores, or for maintenance of spindle polarity, etc, etc. Along the same lines, it's hard to rule out that some defects in mitosis might not derive from DNA-associated functions of these proteins, since if DNA is not properly tightly packaged into prophase chromosomes one would also expect defects in cell cycle progression and DNA damage. I think this ambiguity could be cleared up considerably if the authors undertook a basic structure/function analysis of CHD2, in which they make deletion

© 2023. Published by The Company of Biologists under the terms of the Creative Commons Attribution License (https://creativecommons.org/licenses/by/4.0/).

constructs for the chromodomains, helicase domains, and DNA binding domains, and ask which of these still result in localization to MTs. A more rigorous analysis would also test the functionality of each construct.

The main advance to the field that this manuscript provides is expanding the possible relevant functions of these proteins beyond gene expression regulation. The ASD field has been pouring time and resources into ChIP-Seq, ATAC-Seq, and RNA-Seq experiments for the past 10 years, without identifying robust transcriptional targets for even the most well-studied ASD genes like CHD8 (Wade et al 2019; PMID: 30692911). Given our previous work showing that many ASD genes are required for NPC development, we think this potential convergent function in mitosis, with the acknowledgement that different chromatin regulators may act at different points during mitosis, is a major advance and suggests that we should be doing experiments beyond looking at gene expression regulation for these proteins. We have addressed this point in our discussion and highlight the need for detailed mechanistic work for each one of these proteins, as they may act in different ways and at different points during mitosis. The key point here, though, is that their convergent function in ASD may not be at the level of gene expression regulation. The scenario you suggest is possible and should be explored.

We also appreciate the suggestion of a structure-function analysis, but have concerns about interpreting these results since even DNA-binding domains are known to be required to target some proteins to microtubules (Haque et al 2022, PMID: 35725768). Similarly, importins, Ran, and associated nuclear-localization sequences (NLSs) are involved in mitotic spindle formation (Natchury et al., Cell 2001; PMID: 11163243). Indeed, NLSs are known to sequester spindle assembly factors in the nucleus during interphase to avoid premature spindle formation (a nice summary is in the introduction of Ciciarello et al., 2004; PMID: 15572412). There appears to be an intricate link between nuclear and spindle targeting, and mutants or deletion constructs may not be able to discriminate these functions. Therefore, if we see phenotypes or mislocalizations of DNA-binding domain mutants, it may still be because of a spindle-related function. In future work, we plan to use the *Xenopus* egg extract system (where spindles can form in the absence of nuclei) to be able to separate these functions, but believe this fine mechanistic work to be beyond the scope of this manuscript.

3. A more minor point, but still on this theme, is that I think the authors' speculation that CHD2 functional helicase activity but not DNA binding is required for spindle localization (lines 163-164) is premature at this stage. Right now all we know is that one point mutation changes localization and the other does not. But a structure function analysis of CHD2 as proposed above would also address this concern.

We have removed this sentence.

Reviewer 3
Advance Summary and Potential Significance to Field:
These authors had previously identified evidence for the involvement of microtubule dysfunction in ASD.

In this work they first show that five chromatin regulators most strongly associated with ASD (ADNP, CHD8, CHD2, POGZ, and SUV420H1/KMT5B) might play a role in microtubule biology. They found that all five localized to microtubules of the mitotic spindle in in both Xenopus embryos and in human HEK293 cells. They next asked if CHD2 loss-of-function would lead to defects in mitosis. They inhibited CHD2 expression via in human induced pluripotent stem cell (iPSC) derived cortical neural progenitor cells using CRISPRi and observed a modest increase in abnormal mitotic spindles. They also found that CHD2 reduction led to increased Cyclin B and decreased Cyclin E consistent with activation of a mitotic spindle checkpoint. They also observed an increase in pH2AX puncta, consistent with DNA damage, and an increase in cell death. Finally they generated ASD-associated missense mutations in CHD2, expressed them in Xenopus and found that one of these, CHD2D856G failed to localize to the mitotic spindle.

© 2023. Published by The Company of Biologists under the terms of the Creative Commons Attribution License (https://creativecommons.org/licenses/by/4.0/).

Interestingly this mutation lies within the helicase domain. Overall this is a brief but interesting research report. It would be appropriate for publication in development following revisions to address a few points.

We thank the reviewer for their close reading and positive outlook on publication.

Comments for the Author:
1) The authors should further address whether CHD2 localizes to mitotic spindles across stages and cell types. They look at HEK293 cells and also epidermis of Xenopus tailbud stage embryos. The latter seeming an odd choice - if one was going to look at epidermis one could do so at earlier stages. Does CHD2 localize to the spindle of early embryonic blastomeres and does it in neural progenitor cells as later stages?

We have added several new experiments to the revised manuscript to address this comment. In the revised manuscript we now show endogenous localizations for all 5 chromatin factors on mitotic spindles in early *Xenopus* embryonic blastula cells (Figures S3C). We also validate the CHD2 antibody *in vivo* by injecting *chd2* antisense oligonucleotides and Western blot (Figure S3A-B). We also include these antibody stainings in human iPSC-derived NPCs (Figure S3D) and human iPSC-derived neurons (Figure S3E). In the NPCs, during interphase there is signal within the nucleus (as expected), and during M phase the signal is very diffuse, but there is some overlap with the microtubules of the spindle. In the neurons, there is signal within the nucleus (as expected), but also along the microtubules of the axons.

We also expand our analyses of protein-protein interaction data to include centrosomal networks generated from human NPCs and neurons from O'Neill et al (Science 2022; PMID: 35709258). Again, we detect significant enrichment of ASD-associated proteins within these networks (Figure 4, Table S2). Together, there is compelling evidence that ASD-associated proteins, including those annotated as 'chromatin regulators,' localize to microtubule-rich structures endogenously.

2) In examining the localization of the ASD-associated mutations in Figure 3 the authors should also show images for localization in interphase cells. There seems to be a significant increase in association with chromatin for CHD2D856G. It would be good to show more than a single nucleus, show that the three CHD2 proteins are being expressed at equivalent levels, and quantify localization differences.

We have now expanded this figure to include interphase images and a quantification with statistics of the localization differences (Figure 3). We also now provide images of more cells, all imaged with the same settings, showing similar expression level (Figures 3B, S5).

3) It is surprising that loss of function data is only provided for the iPSC-derived cortical neural progenitors not the embryos. These authors have previously published that CRiSPR-mediated knock down of CHD2 leads to decreased telencephalon size. The authors should attempt to rescue this phenotype with wt CHD2 vs CHD2D856G vs CHD2G1174D. This would provide important information on whether is is the DNA binding domain, the helicase domain (and by proxy spindle binding) or both that contribute to the telencephalon phenotype.

The rescue approach described is a powerful strategy to assign functionality to variants. It requires: 1) a strong starting phenotype to rescue, and 2) the wildtype construct robustly rescues. This has worked in our hands in the past for the gene DYRK1A, where the sgRNA was very efficient, the brain size reduction was strong, and overexpression of the wildtype construct alone caused the opposite phenotype (Willsey et al., Development 2020; PMID: 32467234).

For CHD2, our previous work also used a F0 CRISPR-based approach, but with a sgRNA with a relatively low average mutational efficiency of 59% (Willsey et al., Neuron 2021; PMID:33497602). While this sgRNA caused a statistically significant reduction in telencephalon size, the average reduction in size was modest (~10% reduction) and variable between animals since it was an F0 analysis. To screen variants reliably for rescue, one needs a stronger starting phenotype that is less variable. This would require testing several new sgRNAs to find ones that

© 2023. Published by The Company of Biologists under the terms of the Creative Commons Attribution License (https://creativecommons.org/licenses/by/4.0/).

induce a stronger phenotype more reliably, or potentially switch to a morpholino-based approach, or develop a germline mutant line. Further, the rescue itself is also complicated by the dosage-sensitive nature of CHD2, where increased CHD2 expression also causes a severe developmental disorder and reduced growth (Rom et al, PMID: 31704914). Therefore, we will need to titrate the rescue dose extensively to compensate for the mutation, without generating an over-expression.

While we did attempt all of this for this revision, it became clear to us that these rescue experiments will take longer than an appropriate amount of time for a revision, so we have not been able to include them here.

Second decision letter

MS ID#: DEVELOP/2022/201515

MS TITLE: Pleiotropy of autism-associated chromatin regulators

AUTHORS: Micaela Lasser, Nawei Sun, Yuxiao Xu, Sheng Wang, Sam Drake, Karen Law, Silvano Gonzalez, Belinda Wang, Vanessa Drury, Octavio Castillo, Yefim Zaltsman, Jeanselle Dea, Ethel Bader, Kate E. McCluskey, Matthew W. State, Arthur Jeremy Willsey, and Helen Rankin Willsey

I have now received the reports of two referees who reviewed the earlier version of your manuscript and I have reached a decision. The referees' comments are appended below, or you can access them online: please go to BenchPress and click on the 'Manuscripts with Decisions' queue in the Author Area.

The reviewers' overall evaluation is positive and we would like to publish a revised manuscript in Development. Before this, could you address the remaining comment of Referee 2 to include in the Discussion a comment about the challenges of a structure-function analysis. Please attend to this comment in your revised manuscript and in your point-by-point response. If you do not agree with any of their criticisms or suggestions explain clearly why this is so. If it would be helpful, you are welcome to contact us to discuss your revision in greater detail. Please send us a point-by-point response indicating your plans for addressing the referee's comments, and we will look over this and provide further guidance.

Reviewer 2

Advance summary and potential significance to field

The summary of the advance remains the same as for the initial submission.

Comments for the author

The authors have mostly addressed my previous comments well.
In the case of the proposed structure-function analysis, I appreciate the authors' thoughtful discussion that disentangling DNA and microtubule binding regions may not be so straightforward, as precedent in other proteins suggest an "intricate link between nuclear and spindle targeting, and mutants or deletion constructs may not be able to discriminate these functions." This being the case, I suggest that the authors ensure those precedents and considerations are included in the Discussion, since they will help address to readers of the finished manuscript why such an analysis was beyond the scope of the work, and will help lay out the considerations for later work that may attempt to better resolve the multiple potential functions of these proteins.

© 2023. Published by The Company of Biologists under the terms of the Creative Commons Attribution License (https://creativecommons.org/licenses/by/4.0/).

Reviewer 3

Advance summary and potential significance to field

These authors had previouslyidentified evidence for the involvement of microtubule dysfunction in ASD. In this work they first show that five chromatin regulators most strongly associated with ASD (ADNP CHD8, CHD2, POGZ, and SUV420H1/KMT5B) might play a role in microtubule biology. They found that all five localized to microtubules of the mitotic spindle in in both Xenopus embryos and in human HEK293 cells. They next asked if CHD2 loss-of-function would lead to defects in mitosis. They inhibited CHD2 expression via in human induced pluripotent stem cell (iPSC) derived cortical neural progenitor cells using CRISPRi and observed a modest increase in abnormal mitotic spindles. They also found that CHD2 reduction led to increased Cyclin B and decreased Cyclin E consistent with activation of a mitotic spindle checkpoint. They also observed an increase in pH2AX puncta, consistent with DNA damage, and an increase in cell death. Finally they generated ASD-associated missense mutations in CHD2, expressed them in Xenopus and found that one of these, CHD2D856G failed to localize to the mitotic spindle. Interestingly this mutation lies within the helicase domain.

Comments for the author

In this revised manuscript the authors have sufficiently address the concerns of all three reviewers and I strongly support its publication.

Second revision

Author response to reviewers' comments

Dear Reviewers,

Thank you for your timely and thoughtful feedback. See below **in blue** for our responses to your comments. We appreciate your help improving the manuscript.

Best,
Helen

Reviewer 2 Advance Summary and Potential Significance to Field:
The summary of the advance remains the same as for the initial submission.

Reviewer 2 Comments for the Author:
The authors have mostly addressed my previous comments well. In the case of the proposed structure-function analysis, I appreciate the authors' thoughtful discussion that disentangling DNA and microtubule binding regions may not be so straightforward, as precedent in other proteins suggest an "intricate link between nuclear and spindle targeting, and mutants or deletion constructs may not be able to discriminate these functions." This being the case, I suggest that the authors ensure those precedents and considerations are included in the Discussion, since they will help address to readers of the finished manuscript why such an analysis was beyond the scope of the work, and will help lay out the considerations for later work that may attempt to better resolve the multiple potential functions of these proteins.

We have addressed this comment in the discussion, which now reads: “Given the precedent for chromatin regulators (including CHD3 and CHD4) to play independent and direct roles at both chromatin and microtubules, it is likely that these functions are separable. However, testing this is made difficult by the precedent for DNA-binding domains and nuclear localization sequences to function in microtubule targeting (Haque et al. 2022; Nachury et al. 2001; Gruss et al. 2001), so deletion constructs in these domains may not be able to discriminate between these functions. Rather, these functions could be separated in the powerful *Xenopus* cell-free cytoplasmic egg extract system, where mitotic spindles can form in

© 2023. Published by The Company of Biologists under the terms of the Creative Commons Attribution License (https://creativecommons.org/licenses/by/4.0/).

the absence of nuclei (Heald et al. 1996; Gard and Kirschner 1987). This will be important future work."

Reviewer 3 Advance Summary and Potential Significance to Field:
These authors had previously identified evidence for the involvement of microtubule dysfunction in ASD. In this work they first show that five chromatin regulators most strongly associated with ASD (ADNP, CHD8, CHD2, POGZ, and SUV420H1/KMT5B) might play a role in microtubule biology. They found that all five localized to microtubules of the mitotic spindle in in both Xenopus embryos and in human HEK293 cells. They next asked if CHD2 loss-of-function would lead to defects in mitosis. They inhibited CHD2 expression via in human induced pluripotent stem cell (iPSC) derived cortical neural progenitor cells using CRISPRi and observed a modest increase in abnormal mitotic spindles. They also found that CHD2 reduction led to increased Cyclin B and decreased Cyclin E consistent with activation of a mitotic spindle checkpoint. They also observed an increase in pH2AX puncta, consistent with DNA damage, and an increase in cell death. Finally they generated ASD-associated missense mutations in CHD2, expressed them in Xenopus and found that one of these, CHD2D856G failed to localize to the mitotic spindle. Interestingly this mutation lies within the helicase domain.

Reviewer 3 Comments for the Author:
In this revised manuscript the authors have sufficiently address the concerns of all three reviewers and I strongly support its publication.

Thank you.

Third decision letter

MS ID#: DEVELOP/2022/201515

MS TITLE: Pleiotropy of autism-associated chromatin regulators

AUTHORS: Micaela Lasser, Nawei Sun, Yuxiao Xu, Sheng Wang, Sam Drake, Karen Law, Silvano Gonzalez, Belinda Wang, Vanessa Drury, Octavio Castillo, Yefim Zaltsman, Jeanselle Dea, Ethel Bader, Kate E. McCluskey, Matthew W. State, Arthur Jeremy Willsey, and Helen Rankin Willsey
ARTICLE TYPE: Research Report

I am delighted to tell you that your manuscript has been accepted for publication in Development, pending our standard ethics checks.

© 2023. Published by The Company of Biologists under the terms of the Creative Commons Attribution License (https://creativecommons.org/licenses/by/4.0/).
